# 

**Published:** 1882-08

**Authors:** 


CONTENTS.

| | Page. |
|---|---|
| Autumnal Diseases | 403 |
| Washing | 404 |
| Feeding the Sick | 405 |
| Cold in the Head | 407 |
| Dirt | 407 |
| Is Consumption Contagious ? | 408 |
| Table Manners | 409 |
| Tomatoes | 410 |
| Slandering Doctors | 411 |
| Science Aiding Justice | 411 |
| The Best Inheritance | 415 |
| Science and Christianity | 415 |
| Living Beyond One's Means | 419 |
| Hearty Suppers | 422 |
| Mental Rest | 425 |
| Eating and Drinking | 428 |
| Sunshine | 428 |
| Premature Deaths | 431 |
| Success in Life | 431 |
| Inhalation and Consumption | 433 |

